# Antiplatelet antibodies in cases of Glanzmann's thrombasthenia with and without a history of multiple platelet transfusion

**DOI:** 10.4103/0971-6866.50866

**Published:** 2009

**Authors:** Kanjaksha Ghosh, B. Kulkarni, S. Shetty, S. Nair

**Affiliations:** Institute of Immunohaematology (ICMR), KEM Hospital, Parel, Mumbai, India

**Keywords:** Antiplatelet antibodies, Glanzmann's Thrombasthenia, Flow cytometry

## Abstract

Antiplatelet antibodies are known to be present in a wide spectrum of patients, which include chronic Idiopathic Thrombocytopenic Purpura (ITP), infections, etc., including Glanzmann's thrombasthenia (GT) patients who receive multiple platelet transfusions. The presence of natural antibodies to platelet receptors is not studied in cases of GT. We studied the antiplatelet antibodies in 23 patients with GT, 15 of which had received multiple transfusions and eight that had not received transfusions, along with 50 cases of chronic ITP. The prevalence and specificity of platelet-bound antibodies were detected by inhibition assays using O-group platelets on flow cytometry. The mean antiplatelet antibodies in 15 patients of GT who had not received transfusions and eight patients with multiple transfusions was 8427 + 2131.88 and 9038 + 2856 antibodies/platelet, respectively, while in case of the 50 ITP patients studied, it was 22166 + 5616 antibodies/platelet (Normal Range 1500–3200 antibodies/platelet). We conclude that GT patients who have not received transfusions may develop antiplatelet antibodies to the missing/abnormal receptor. Whether this is due to a molecular mimicry or due to some other mechanism needs to be explored.

## Introduction

Immune recognition to self-antigens is obtained in early fetal life *in utero*. The immune memory recognizes these self-antigens and does not elicit an immune response to them. A classical example of this phenomenon is the blood group agglutinins. However, antibodies may be produced against some such self-antigen epitopes because of a molecular mimicry with some of the natural infectants (virus or bacteria) or intestinal microorganisms. In the early 1980s’, Caen *et al.* demonstrated the presence of antibodies formed against the glycoprotein receptor IIb/IIIa on the human platelet surface membrane,[1] which was congenitally lost in cases of Glanzmann's thrombasthenia (GT). GT is an inherited disorder of the platelet surface membrane glycoprotein IIb/IIIa complex. It is an autosomal recessive disorder of platelet function that produces a lifelong bleeding tendency, caused by deficiency or abnormality of the membrane glycoprotein GP-IIb/IIIa complex. This results in bleeding due to defective platelet hemostatic plug formation. These patients require platelet transfusions when they have a bleeding episode. Some such multitransfused GT patients become clinically refractory to platelet transfusions.[2,3] It is not known whether GT patients who have not received any platelet or blood transfusions show the presence of antibodies that may be produced as a result of a molecular homology to some of the common intestinal microbes or natural bacterial or viral infections in the same way as natural blood group antibodies are formed. It is known that in cases of ITP, antiplatelet antibodies are produced, which may have specificity for either GPIIb/IIIa or Ib/IX complexes.[4] GT is a rare disorder and hence this was a unique opportunity to investigate these patients, both multitransfused and ones who were never transfused with blood or platelet concentrates.

## Materials and Methods

**Figure 1:**
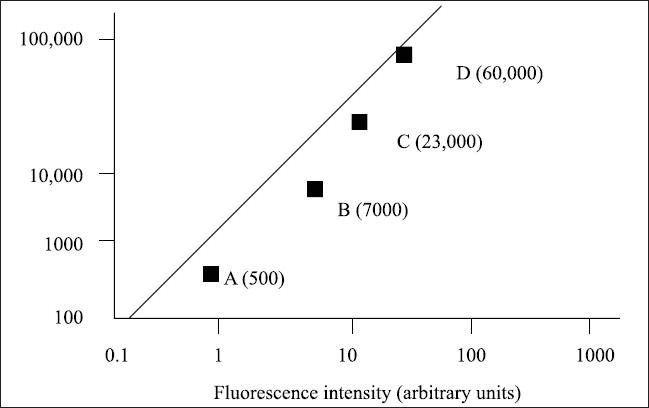
Calibration curve for platelet antibodies by flow cytometry MAb Molec/platelet

Fifteen patients of GT who had not received any transfusions and eight patients of GT with a history of multiple platelet transfusions were studied. Fifty cases of chronic ITP were also included in this study. FITC-labeled rabbit anti-human immunoglobulin (Ig) G (Dako cytomation, Glostrup, Denmark) was used for the determination of antiplatelet antibodies. Rabbit anti-mouse IgG-FITC was used as the negative control and mouse anti-human GPIIb/IIIa and GPIb/IX were used for the receptor studies. The receptor studies in the GT patients were carried out using the patient platelets and FITC-tagged receptor-specific antibodies (Dako cytomation, Glostrup, Denmark, and BD Biosciences, San Jose, CA, USA). For the determination of platelet-bound antibodies, patient serum (1:5) was incubated with normal O-group platelets at room temperature for 30 min. After washing thrice in buffered citrated saline, these platelets were incubated with FITC-anti-human globulins for 20 min at room temperature and the fluorescence was measured as mean fluorescence intensity (MFI) by flow cytometry. For the quantitation of antibody, a suspension of calibrated beads precoated with increasing and accurate quantities of Igs was used (Diagnostica Stago, Asnieres, France). These beads were incubated with F(ab’)_2_ polyclonal anti-IgG-FITC for 20 min and subjected to flow cytometry. The MFI values of samples and controls were extrapolated on the calibration curve and their corresponding molecule numbers were read directly.[5] Flow cytometry analysis was performed on a BD FACS Calibur Flow cytometer. The dual scatter dot plot was set in logarithmic coordinates (FS Log X SS Log). The MFI values were plotted on a Log-Log graph paper [Figure 1].

### Human platelet antigen-1 (HPA-1) genotyping

HPA-1 genotyping was carried out using allele-specific polymerase chain reaction[6] in GT cases with milder bleeding diathesis.[7]

## Determination of specificity of antiplatelet antibody to GPIIb/IIIa complex in GT serum

A pool of platelets from five O Rh-positive normal healthy subjects was taken. The platelets were washed and the GPIIb/IIIa receptor occupancy was studied using FITC-tagged mouse anti-human GPIIb/IIIa and GPIb/IX antibodies (BD Biosciences and Dako Cytomation) by flow cytometry. The washed platelets were incubated with GT patient serum (1:5) for 30 min at room temperature. Percent inhibition of fluorescence-tagged GPIIb/IIIa or GPIb/IX antibody binding was noted by flow cytometry. O-group-normal platelets were incubated for 30 min at room temperature with GT patient sera (1:5) and then washed thrice to remove unbound excess antibodies. [Table 1a and 1b] shows the inhibition of fluorescence to GPIIb/IIIa receptors in the presence of GT sera in both transfused and nontransfused patients with GT.

**Table 1a:** Inhibition of fluorescence to IIbIIIa in the presence of nontransfused GT patient sera

Reaction	Residual IIbIIIa%	Residual IbIX%	GPIIbIIIa % inhibition	GPIbIX % inhibition	Antibody/platelet
O-group platelets	87.26 ± 6.34	86.12 ± 8.04	0	0	1800 ± 223.88
O-group platelets + normal sera (1:5)	81.33 ± 4.12	82.56 ± 5.66	6.89 ± 6.1	4.13 ± 5.74	2300 ± 316.54
S	15.53	81.09	87.79	9.42	11,000
GK	0	82	83.65	7.91	9000
MK	0	86	84.17	3.01	9500
T	0	87	89.55	6.97	12,000
VS	0	90	84.05	14.07	10,000
GR	1	90	83.8	9.93	9000
P	0	80	82.27	8.02	9000
AK	2.57	94.3	83.74	3.64	10,000
HD	4.29	94.29	83.69	6.72	6000
SS	0	80	78.61	7.1	4900
VW	95.39	84.36	84.58	8.6	9000
MP	0	81	74.83	5.15	5500
AC	3.46	86.12	76.69	10.35	6500
AK	8.9	80.12	74.6	7.21	6000
RS	0.3	97.89	82.66	4.5	9000
Mean of patients	8.762	86.278	82.312	7.506	8427
Standard deviation of the patients	24.357	5.119	4.332	2.844	2131.889

**Table 1b:** Inhibition of fluorescence to IIbIIIa in the presence of transfused GT patient sera

Reaction	Residual IIbIIIa%	Residual IbIX%	GPIIbIIIa % inhibition	GPIbIX % inhibition	Antibody platelet
O-group platelets	87.26 ± 6.34	86.12 ± 8.04	0	0	1800 ± 223.88
O-group platelets	87.26 ± 6.34	86.12 ± 8.04	0	0	1800 ± 223.88
O-group platelets + normal sera (1:5)	81.33 ± 4.12	82.56 ± 5.66	6.89 ± 6.1	4.13 ± 5.74	2300 ± 316.54
YS	52.4	64.4	89.44	7.9	15,000
AL	0	98	83.11	5.73	8000
CL	0	89	81.75	8.48	6800
DS	0	85	84.59	4.63	10,000
SM	93	89	87.21	9.98	11,000
M	10.69	81.54	81.02	7.08	7000
KP	20.06	75.63	80.49	9.21	7000
SP	24.1	78	80.51	9.85	7500
Mean of patients	25.031	82.571	83.515	7.857	9038
Standard deviation of the patients	32.688	10.190	3.329	1.936	2856

## Results and Discussion

All the 15 GT patients not having received platelet transfusions showed the presence of antiplatelet antibodies (8427 + 2131.88, NR: 1500–3200 abs/platelet). The absorption of antibodies against normal pooled platelets reduced the GPIIb/IIIa expression by 82.31 ± 4.33% in all the GT patients, showing that the major determinant against which the antibodies were directed in the GT sera was the GPIIb/IIIa complex, whereas in chronic ITP patients, this inhibition was variable.[8] The group of eight patients of GT with history of multiple platelet transfusions also showed the presence of antiplatelet antibodies (9038 + 2856, NR: 1500-3200 abs/platelet) directed mainly toward the GPIIb/IIIa complex. In the 50 ITP patients studied, it was 22166 + 5616 antibodies/platelet. Two of the 15 patients not having received platelet transfusions were found to be homozygous for HPA-1b allele. Most of the patients recruited in this study were pediatric patients (range: 2–34 years, mean age: 11.17 + 7.93 years). None of the female patients had any history of pregnancy. Of the eight transfused patients, four were male and four were female and of the 15 non-transfused patients, four were male and 11 were female. [Table  1a and 1b] the residual receptors and the antibodies in GT patient sera that bound to normal O-group platelets, both in the transfused and the nontransfused groups of patients.

The receptor studies in the GT patients were carried out using the patient platelets and FITC-tagged receptor-specific antibodies (BD Biosciences and Dako Cytomation). Estimation of platelet antibodies was performed conventionally using enzyme-linked immunosorbent assay (ELISA)-based assays, which give the measure of antibodies as PAIgG (ng/10^6^ platelets),(8) whereas by flow cytometry, the measure obtained is antibody/platelet. Comparison studies for the determination of platelet antibodies by ELISA-based assays and immunofluorescence showed that the fluorescence method had a high sensitivity in the detection of platelet antibodies than PAIgG by ELISA-based assays.[5] The specificity of the antibodies to platelets in GT patient sera was found to be mostly directed toward the GPIIb/IIIa receptors, as observed from the inhibition studies [Table 1a and 1b].

GT patients who receive repeated platelet transfusions are known to show the presence of antiplatelet antibodies directed against the glycoprotein receptors.[1–3] However, it was observed in the present study that the GT patients without a history of platelet transfusions or any blood product transfusion also showed the presence of antibodies directed against the GPIIb/IIIa complex, which is absent in most of these patients. In the group of GT patients who had not received platelet transfusions and were clinically mild on presentation, two patients were found to be homozygous for the HPA-1b allele, which is reported to have more affinity for fibrinogen binding to the GPIIb/IIIa receptors.[9,10] It has been reported that platelet-reactive autoantibodies can also bind to the mitochondrial antigen M2(10) and dengue viral nonstructural protein 1 (NS1), which exhibits a tendency to elicit potentially hazardous antibodies thus showing a wide spectrum of specificity against extracellular matrix and platelet antigens as well.[12] These findings suggest that GT patients without a history of transfusions with blood products may produce antibodies to the platelet glycoprotein receptor IIb/IIIa, which is congenitally absent in these patients, in the same manner as anti-A agglutinin is produced in the B-positive blood group patients and anti-B agglutinin is produced in the A-positive blood group patients. For the ABO blood group system, it has been firmly established that these agglutinins, which are called natural antibodies, develop because of exposure to similar antigens in the bowel microflora of the individual. Using similar analogy, we searched all the Pubmed articles using key words like “antibodies,” “antiplatelet antibodies,” “natural antibodies to platelets,” “primary platelet refractoriness,” and “Glanzmanns thrombasthenia” from 1960 to 2006. We also looked for sequence homology between platelet membrane glycoproteins and bacterial or viral peptides. We found reports suggesting sequence homologies between the platelet membrane glycoproteins and mitochondrial antigen M2,[11] dengue viral NS1,[12] and human immunodeficiency virus-1 GP41.[13] It may not be out of place to mention here that dengue and other arbovirus infections are very common in India in addition to ubiquitous Ebstein-Barr virus and cytomegalovirus, which are known to cause immune thrombocytopenia by unknown mechanisms. Some of our patients who developed anti-GPIIb/IIIa antibodies had higher levels of GPIIb/IIIa on their platelet surface by flow cytometry. These patients probably have a missense mutation, which allowed an altered nonfunctional protein to be expressed on the platelet surface as a self-antigen, leading to the emergence of antibody against the normal nonself GPIIb/IIIa antigen, which could be picked up by incubation with normal GPIIb/IIIa-bearing O Rh-positive platelets.

In conclusion, it may be said that severe GT patients may spontaneously develop antiplatelet antibodies directed to the GPIIb/IIIa complex and that their platelet counts remain normal, in contrast to what happens to many patients with chronic or acute ITP where antibodies may be directed to the GPIIb/IIIa complex. In case of ITP, this antibody is considered as an autoantibody whereas in GT patients, this is an alloantibody in nature. Whether antigenic mimicry to some bacterial or viral peptides, which are common in our environment, is the cause for this finding needs to be determined. It also makes it plausible to face a patient of severe GT who may show primary refractoriness to platelet transfusion, although we did not find any reference to that effect in the English literature.
